# Achieving Partial Nitrification-Anammox Process Dependent on Microalgal-Bacterial Consortia in a Photosequencing Batch Reactor

**DOI:** 10.3389/fbioe.2022.851800

**Published:** 2022-03-18

**Authors:** Miao Yang, Kun-Peng Xie, Chi Ma, Si-Hui Yu, Jing-Yi Ma, Ze-Quan Yu, Xi Chen, Zheng Gong

**Affiliations:** ^1^ Key Laboratory of Plant Biotechnology of Liaoning Province, School of Life Sciences, Liaoning Normal University, Dalian, China; ^2^ Dalian Key Laboratory of Marine Bioactive Polypeptide Drugs, School of Life Sciences, Liaoning Normal University, Dalian, China

**Keywords:** biological nitrogen removal, microalgal–bacterial consortia, photosequencing batch reactor, microbial community, anammox, algammox biofilm system

## Abstract

Partial nitrification coupled with anammox (PN/A) process is an energy-efficient approach for nitrogen removal from low C/N wastewater. In this study, PN/A was achieved with optimal oxygen supply from a green microalga, *Chlorella sorokiniana*. The PN process was first initiated within 35 days, and the following algae-intensified PN then reached the steady state within the next 32 days. The dissolved oxygen (DO) concentration was gradually maintained at 0.6 mg L^−1^ *via* adjusting the photoperiod to 6-h light/18-h dark cycles, when the accumulation ratio of NO_2_^−^-N and the removal ratio of NH_4_^+^-N were both more than 90%. The nitrogen removal capability of anammox was acclimated *via* elevating the individual effluent NH_4_^+^-N and NO_2_^−^-N levels from 100 to 200, to 300 mg L^−1^. After acclimation, the removal rates of NH_4_^+^-N and total nitrogen (TN) reached more than 70 and 80%, respectively, and almost all the NO_2_^−^-N was removed. Then, the algae-intensified PN/A, algammox biofilm system, was successfully started up. When the NH_4_^+^-N level increased from 100 to 300 mg L^−1^, the TN removal varied between 78 and 82%. In the photosequencing bioreactor, *C. sorokiniana*, ammonia-oxidizing bacteria (AOB), and anammox coexisted with an illumination of 200 μmol m^−2^ s^−1^ and a 6-h light/18-h dark cycles. The DO levels ranged between 0.4 and 0.5 mg L^−1^. In addition, the microbial community analysis by Illumina MiSeq sequencing showed that the dominant functional bacteria in the algae-intensified PN/A reactors included *Nitrosomonas* (AOB) and *Candidatus Brocadia* (anammox), while *Nitrospira* and *Nitrobacter* (nitrite oxidizing bacteria), together with *Denitratisoma* (denitrifier) were largely inhibited. Further studies are required to optimize the microalgal–bacterial consortia system to achieve superior nitrogen removal rates under controllable conditions.

## Introduction

High-strength ammonium wastewater has greatly threatened water preservation and human health and caused severe environmental issues due to the cytotoxicity of free ammonia to aquatic organisms (Wang et al., 2015), as well as excessive algal growth induced by eutrophication (Conley et al., 2009). The industrial wastewater from food, fertilizer, plastics, petrochemical and pharmaceutical industries; the centrate from anaerobic digestion of domestic, agricultural, and industrial wastes; and landfill leachate are all ammonium-abundant wastewater (Carrera et al., 2003; Fan et al., 2020).

Currently, the ammonium-rich wastewater is usually treated *via* biological nitrogen removal (BNR) processes, and conventional BNR is performed by aerobic nitrification and anaerobic denitrification, involving ammonium oxidizing bacteria (AOB), nitrite oxidizing bacteria (NOB), and denitrifiers (Winkler and Straka, 2019). However, nitrification requires energy intensive aeration, while denitrification requires organic carbon as the external electron donor, to assure effective removal of nitrogen (Sun et al., 2010; McCarty et al., 2011). To reduce oxygen and organic carbon demands, shortcut nitrogen removal *via* nitrite process has been developed to treat ammonium-rich wastewater, and it needs inhibiting oxidation of nitrite to nitrate on conditions that activate AOB over NOB (Fux et al., 2006). Moreover, the anaerobic ammonium oxidation (anammox) process is a cost-effective as well as low energy-consuming alternative to the conventional BNR processes, in which ammonium and nitrite are directly oxidized to nitrogen gas under anoxic conditions (van Dongen et al., 2001). One-stage partial nitrification and anammox (PN/A), e.g., completely autotrophic nitrogen removal over nitrite, is a completely autotrophic process and conducted in one single reactor (Sliekers et al., 2002). This process only needs limited oxygen and requires no additional external carbon source, which is an efficient, energy-saving, and environment-friendly biological nitrogen removal technology.

Commonly, the BNR processes dependent on these functional bacteria are difficult to completely eliminate nitrogen, and the microalgal–bacterial consortia has attracted intensive interests due to its various advantages in wastewater treatment (Zhang et al., 2021). On the one hand, microalgae perform photosynthesis to release sufficient oxygen, which is needed for AOB (Mukarunyana et al., 2018; Rada-Ariza et al., 2017). On the other hand, microalgal cells are excellent in nutrient removal and could further boost nitrogen removal effectively (Zhang et al., 2021). Besides, the versatile microalgae are usually rich in multiple natural bioactive components, and their valuable biomass could be used as raw materials for recycling resources (Liu and Hong, 2021). Among the reported microalgal species for wastewater treatment, *Chlorella* has been shown to have superior performance in CO_2_ sequestration and nitrogen removal (Chai et al., 2021; Fallahi et al., 2021; Peter et al., 2021; Zhang et al., 2021). Moreover, it is resilient to variable environmental conditions, and these algal cells could accumulate substantial amounts of lipids, serving as potential candidates for sustainable biofuel production (Ray et al., 2022).

Recently, the completely autotrophic algammox (algal anaerobic ammonium oxidation) system has been developed to integrate microalgae, AOB, and anammox in a photosequencing batch reactor (PSBR), lasting for a total of 30 days (Manser et al., 2016). In the microalgal–bacterial consortium, the oxygen is sufficiently provided *via* microalgal photosynthesis during illumination, substituting conventional mechanical aeration. The influent ammonia was oxidized to nitrite during light (aerobic) periods, followed by anammox-directed consistent reduction of nitrite and ammonium during dark (anoxic) periods. However, the strain information and culture conditions for microalgae were not mentioned in this study. Furthermore, the research team detected impacts of short-duration dissolved oxygen (DO) increase on anammox activity in the algammox system, but the regulation of DO levels was adjusted with the aid of an aquarium aerator, rather than modulating the light cycle for microalgal photosynthesis (Mukarunyana et al., 2018). In addition, these studies did not monitor the microbial community structure in the reactors, and the evidence for functional bacteria to remove nitrogen also turned out to be scarce.

In this work, we integrate a microalgal–bacterial consortium to selectively conduct nitritation with anammox for efficient ammonium removal. No external organic carbon was provided to inhibit denitrification, and no mechanical aeration was also used for this autotrophic process. The PN/A system coupled with the green alga *C. sorokiniana* was successfully started up within 127 days, benefiting from gradual modulation of the light cycle to maintain an optimal DO level. The bacterial community structures in the consortia were investigated to appreciate the PN/A process in the reactors.

## Materials and Methods

### Bioreactor Set-Up and Operation Strategy

**FIGURE 1:**
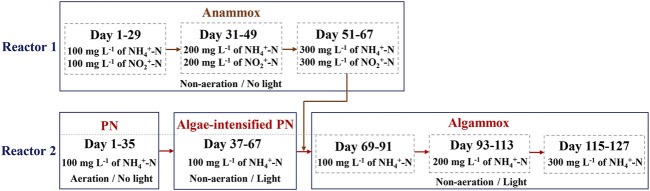
Operation strategy for nitrogen removal. PN denotes partial nitrification.

The operation strategy for nitrogen removal was designed as shown in Figure 1. The PN reactor (Supplementary Figure S1) was initiated first, followed by the startup of the microalgae-intensified PN reactor (Supplementary Figure S2). After the enriched anammox (Supplementary Figure S3) could stably remove the NH_4_^+^-N and NO_2_^−^-N, they were inoculated into the algae-intensified PN reactor. The PN reactor, microalgae–PN reactor, and microalgae–PN coupled with anammox reactor were all equipped with a string of circular nonwoven fabric materials distributed in the center of the reactors, and the hydraulic retention time (HRT) was set as 24 h. A total of eight slices of circular nonwoven fabric discs (diameter 15 cm, thickness 2 cm) were uniformly fixed in the vertical agitator. The cylinder reactor was made by organic glass, and the volume was 7.5 L (inner diameter, 20 cm; height, 50 cm). Oxygen was pumped into the PN reactor from the bottom aerator by an air compressor, and the aeration rate was controlled by a rotameter. The agitator rotated the biodisks at 20 rpm, making the oxygen uniformly distributed in the reactors, and the sludge could be efficiently fixed on the nonwoven fabric materials.

The influent water tank was heated to be maintained at 30 ± 0.5°C. The influent pH value was maintained between 7 and 8 *via* the addition of KHCO_3_. The simulated sewage was entered into the reactors aided with a peristaltic pump, and the effluents were discharged from the upward side of the reactors. The water was successively pumped into and flowed out of the reactors. The initial NH_4_^+^-N concentration for the PN reactor was set at 100 mg L^−1^. Four light tubes (length 60 cm, 20 W) were evenly distributed around the microalgae-intensified PN reactor (Supplementary Figure S2), and the light intensity was 200 μmol m^−2^ s^−1^, which was determined inside the empty reactor. When the microalgal cells were inoculated, the aeration was terminated. Mixing was conducted during the whole period.

### Ammonia-Oxidizing Bacteria Enrichment Culture

AOB was enriched from aerobic activated sludge collected from the Dalian–Dongtai Xiajiahe WWTP (Dalian, China). The mixed liquor volatile suspended solids (MLSS) concentration of the inoculated sludge was determined to be 2 g L^−1^, and the inoculation volume was 2 L. Synthetic wastewater was used as influent in this study, and the media (all the units as mg L^−1^) included KHCO_3_ (1,250), KH_2_PO_4_ (25), CaCl_2_·2H_2_O (300), MgSO_4_·7H_2_O (200), FeSO_4_ (6.25), EDTA (15,000), ZnSO_4_·7H_2_O (430), CoCl_2_·6H_2_O (240), MnCl_2_·4H_2_O (990), and CuSO_4_·5H_2_O (250). The NH_4_^+^-N levels used in the synthetic wastewater were set as 100, 200, and 300 mg L^−1^, prepared *via* (NH_4_)_2_SO_4_.

### Microalgal Cultivation and Growth Evaluation

*C. sorokiniana* was obtained from the Key Laboratory of Plant Biotechnology in Liaoning Province (Liu et al., 2017) and cultured using (NH_4_)_2_SO_4_, KH_2_PO_4_, and Dauta medium without nitrogen and phosphorus. The Dauta medium (all the units as mg L^−1^) contains Na_2_EDTA·2H_2_O (1.1), FeSO_4_·7H_2_O (1), Na_2_CO_3_ (5), MgSO_4_·7H_2_O (25), CaCl_2_·2H_2_O (25), H_3_BO_3_ (0.001), MnCl_2_·2H_2_O (0.4), CoCl_2_·6H_2_O (0.01), ZnSO_4_·7H_2_O (0.02), CuCl_2_·2H_2_O (0.02), and Na_2_MoO_4_·2H_2_O (0.041). The concentrations of NH_4_^+^-N and PO_4_^3−^-P were 100 and 40 mg L^−1^, respectively. The algal cells were subcultured in the flasks under continuous illumination (200 μmol m^−2^ s^−1^) at 25 ± 2°C and shaken three times per day. The exponentially growing cells were inoculated into the reactors with a 10-fold (v/v) dilution ratio. The growth of algal cells in the reactors was determined in terms of the chlorophyll *a* (Chl *a*) content. Certain area of the nonwoven fabric discs containing the retained algal cells and certain volume of fluid containing the suspended algal cells were collected to evaluate the algal growth, respectively. The pelleted algal cells were sonicated in ethanol on ice. After the pigment extracts were centrifuged at 4,500 rpm for 5 min, the supernatant absorption was measured spectrophotometrically at 649 and 665 nm. Chl *a* concentration was then calculated according to the protocol of Jespersen and Christoffersen (1987).

### Anammox Enrichment Culture

Anammox was enriched in another cylinder, acrylic plastic reactor with a volume of 7.5 L (inner diameter 20 cm, height 50 cm). The nonwoven fabric materials (thickness 2 cm) were folded into a fan-like shape and vertically inserted into the reactor to immobilize the attached anammox. No agitator was added to assure the ambient environment to be anaerobic. Other conditions were set as the PN reactor.

The anammox bacteria used in this study were obtained from the Key Laboratory of Industrial Ecology and Environmental Engineering in Dalian Technology University and were maintained at the Key Laboratory of Plant Biotechnology in Liaoning Province, Liaoning Normal University. The MLSS concentration of the inoculated anammox sludge was 2 g L^−1^, and the inoculation volume was 2 L in the anammox reactor. The levels of NH_4_^+^-N and NO_2_^−^-N for the influent were both 100, 200, or 300 mg L^−1^.

### Operation of Algae-Intensified Partial Nitrification and Anammox Reactor

The algae-PN and anammox reactor was operated based on the algae-PN reactor following the inoculation of the enriched anammox mentioned above. The MLSS level of the inoculated anammox sludge with 2 L was 3 g L^−1^. The levels of NH_4_^+^-N for the influent were also 100, 200, or 300 mg L^−1^.

### Analytical Methods

The collected influent and effluent samples were passed through a 0.45-μm filter before analysis. The concentrations of NH_4_^+^-N, NO_2_^−^-N, and NO_3_^−^-N were determined according to the standard methods (APHA, 2012). The DO concentration was detected using a digital portable DO meter (STARTER300D, China). The temperature and pH were measured using a digital portable pH meter (Hi98103, China). The nitrite accumulation rate (NAR) was calculated as Con. NO_2_^−^-N/(Con. NO_2_^−^-N + Con. NO_3_^−^-N) × 100%.

The microbial samples in the anammox, algae-intensified PN, and algae-intensified PN/A reactors were collected on Days 67, 67, and 127, respectively, for biological community analysis, and they were named ANA, MA-PN, and MA-PN-ANA, respectively. These samples were analyzed based on high-throughput sequencing on an Illumuna MiSeq Platform (US). The total genomic DNAs was extracted using the OMEGA Soil DNA Kit (M5635-02, Omega Bio-Tek, Norcross, GA, United States), following the manufacturer’s instructions, and stored at −20°C prior to further analysis. The quantity and quality of the extracted DNAs were determined using a NanoDrop NC2000 spectrophotometer (Thermo Fisher Scientific, Waltham, MA, United States) and agarose gel electrophoresis, respectively. PCR amplification of the bacterial 16S rRNA genes' V3–V4 region was performed using the forward primer 338F (5′-ACT​CCT​ACG​GGA​GGC​AGC​A-3′) and the reverse primer 806R (5′-GGACTACHVGGGTWTCTAAT-3′). The PCR components included 5 μL of buffer (5 ×), 0.25 μL of FastPfu DNA polymerase (5 U/μl), 2 μL (2.5 mM) of dNTPs, 1 μL (10 uM) of each forward and reverse primer, 1 μL of DNA template, and 14.75 μL of ddH_2_O. Thermal cycling was performed as follows: initial denaturation at 98°C for 5 min, followed by 25 cycles involving denaturation at 98°C for 30 s, annealing at 53°C for 30 s, and extension at 72°C for 45 s, with a final extension of 5 min at 72°C. PCR amplicons were purified with Vazyme VAHTSTM DNA Clean Beads (Vazyme, Nanjing, China) and quantified using the Quant-iT PicoGreen dsDNA assay kit (Invitrogen, Carlsbad, CA, United States). Then the amplicons were pooled in equal amounts, and paired-end 2 × 250 bp sequencing was operated using the Illumina MiSeq platform at Shanghai Personal Biotechnology Co., Ltd. (Shanghai, China).

## Results and Discussion

### Startup of Partial Nitrification Reactor

**FIGURE 2:**
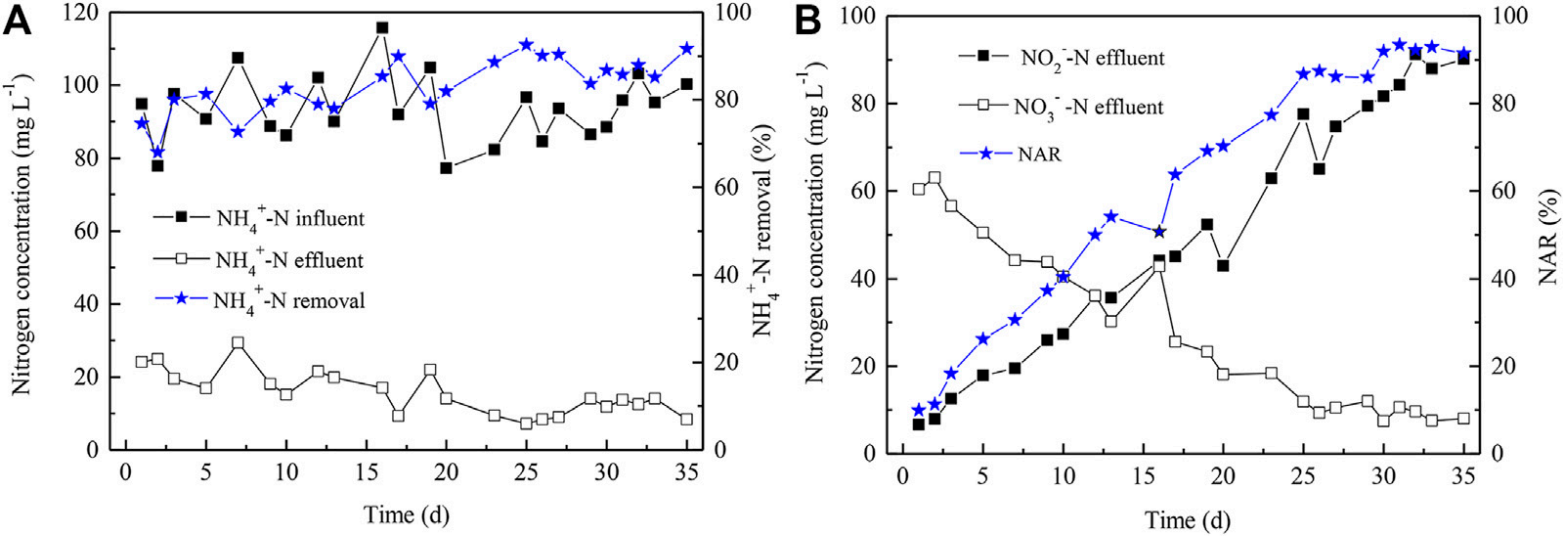
NH_4_^+^-N removal **(A)** and NO_2_^−^-N accumulation **(B)** in the influent and effluent of the PN reactor. NAR indicates nitrite accumulation rate and was calculated as Con. NO_2_^−^-N/(Con. NO_2_^−^-N + Con. NO_3_^−^-N) × 100%.

In order to establish the algammox biofilm system, the PN process was first developed. The successful startup of PN is dependent on constant accumulation of high levels of NO_2_^−^-N, and the NAR needs to achieve more than 50%. Many factors, e.g., temperature, DO, pH, C/N ratio, as well as sludge age, affect nitrite accumulation. In this study, the initial NH_4_^+^-N and DO concentrations were 100 (Figure 2A) and 1.0 mg L^−1^, respectively. The HRT was 24 h and the PN reactor was operated for 35 days (Figure 2). The effluent NAR (Figure 2B) and the NH_4_^+^-N removal efficiency (Figure 2A) both reached up to more than 90% *via* gradual decrease of DO level to 0.6 mg L^−1^. In addition, the effluent NO_3_^−^-N concentration was gradually declined from 61 to 8 mg L^−1^ (Figure 2B), indicating the dominance of AOB, instead of NOB. Thus, the PN process was successfully initiated by gradual reduction of DO levels. The lower nitrate level (8 mg/L) compared to the nitrite level (90 mg/L) in the effluent on Day 35 showed that the high NH_4_^+^-N level, high NO_2_^−^-N level, as well as low DO level co-favored AOB over NOB, though certain NOB appeared to be functioning.

### Startup of Microalgae-Intensified Partial Nitrification Reactor

**FIGURE 3:**
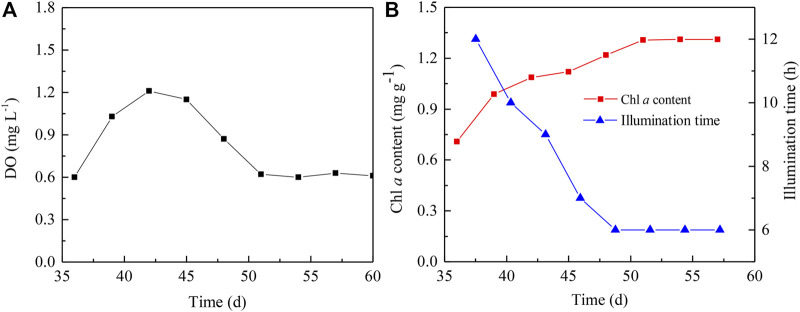
Changes in the DO level **(A)** and Chl *a* content and illumination time **(B)** in microalgae-intensified PN reactor.

The irradiation affects the growth of algal cells, which then determines DO levels in the reactors. The DO levels concern nitrogen removal efficiency of the microalgae-assisted PN reactors. Thus, the light cycle was gradually adjusted to manipulate the DO level in the PN reactors without aeration. The initial light/dark cycles were set as 12 h/12 h, and the DO level gradually increased from 0.6 mg L^−1^ to the highest level, i.e., 1.2 mg L^−1^ (Figure 3A), when the light period was shortened from 12 to 9 h (Figure 3B). During this period, the Chl *a* content in the reactor was augmented by 53% (Figure 3B) mainly due to the exponential growth of the inoculated algal cells, thus accounting for the elevated DO levels accompanied by shortened light periods. When the irradiation time was further shortened to 6 h, the highest DO level began to decrease by approximately 50% along with the continuous increase of Chl *a* content prior to Day 48 (Figure 2). After this, the DO level sustained at 0.6 mg L^−1^ (Figure 3A), and the Chl *a* content was also kept constant at 1.3 mg g^−1^ (Figure 3B).

**FIGURE 4:**
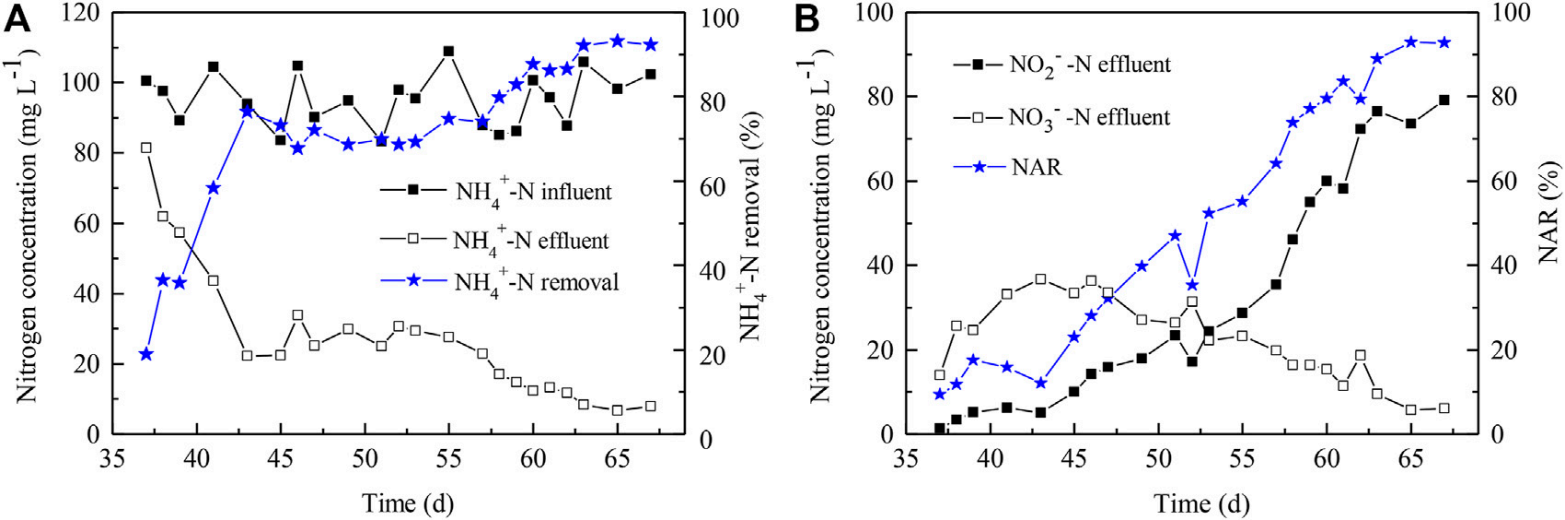
Nitrogen removal performance in the microalgae-intensified PN reactor. **(A)** NH_4_^+^-N removal; **(B)** NO_2_^−^-N accumulation. NAR indicates nitrite accumulation rate and was calculated as Con. NO_2_^−^-N/(Con. NO_2_^−^-N + Con. NO_3_^−^-N) × 100

Microalgae-intensified PN process lasted for 32 days, from Day 35 to 67 (Figure 4). During this period, the NH_4_^+^-N level decreased to 7.99 mg L^−1^ and the NH_4_^+^-N removal achieved up to 92% (Figure 4A); meanwhile, the NAR also increased to 92% (Figure 4B). At this point, the Chl *a* content was maintained at 1.3 mg g^−1^. In addition, the NO_3_^−^-N concentration in the reactor increased first and decreased subsequently; it reached 36.34 mg L^−1^, the highest at Day 46 (Figure 4B). Until Day 53, the NO_2_^−^-N level exceeded the NO_3_^−^-N level (Figure 4B), and the DO level was sustained at 0.6 mg L^−1^ when the light cycles had been adjusted to 6 h light/18 h dark. Thus, it was revealed that the AOB activity indeed surpassed the NOB activity dependent on the available algae-supplied oxygen level, as well as the high level of NO_2_^−^N. AOB became the dominant leader in the algae-intensified PN reactor, though the remaining NH_4_^+^-N and NO_3_^−^-N were still maintained at low levels at the end, which was consistent with the previous reports (Wang et al., 2015; Manser et al., 2016).

### Startup of Anammox Reactor

**FIGURE 5:**
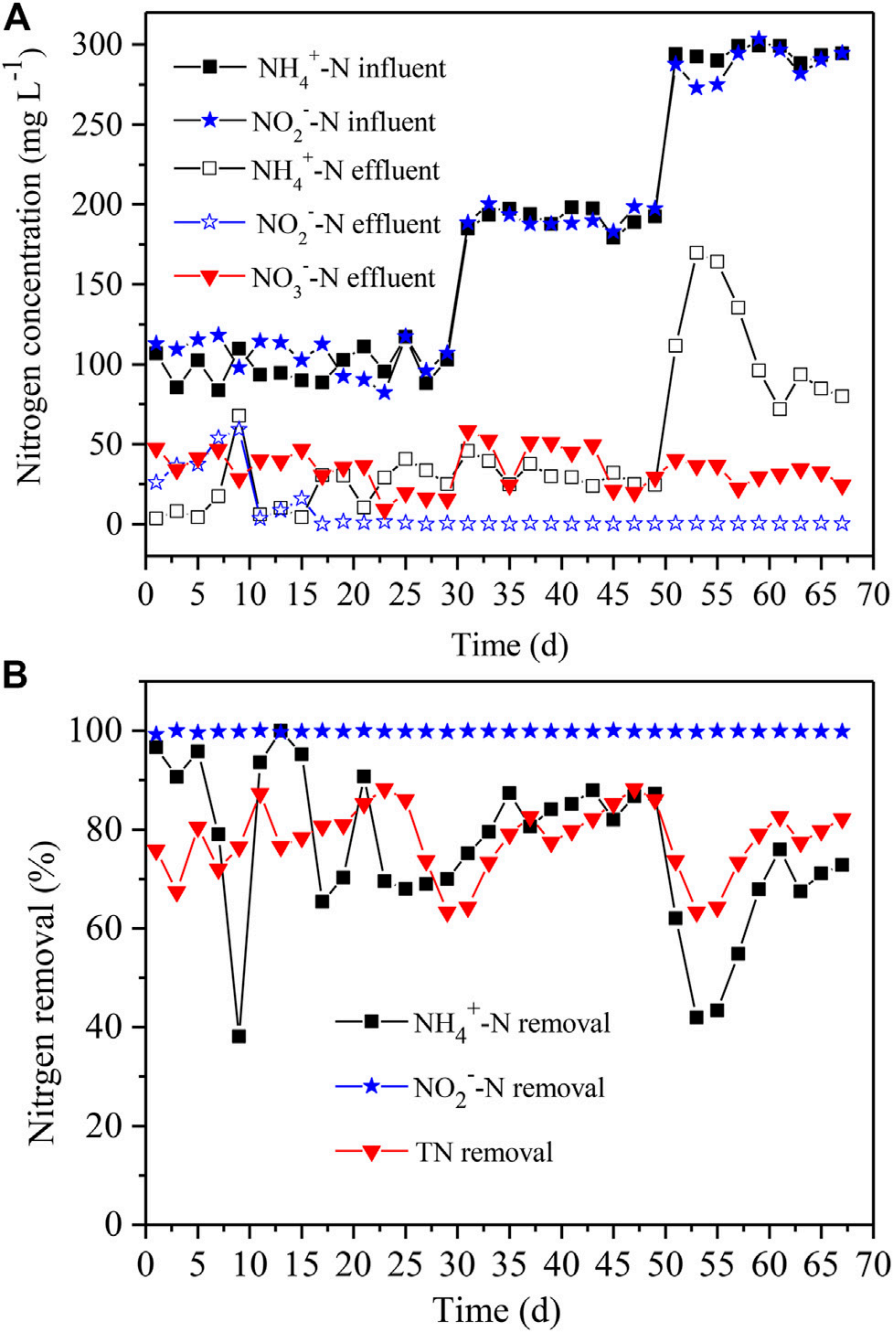
The effluent **(A)** and nitrogen removal **(B)** in the anammox reactor with distinct NH_4_^+^-N and NO_2_^−^-N levels of influent. The NH_4_^+^-N and NO_2_^−^-N concentrations varied from 100, to 200, to 300 mg L^−1^. TN removal denotes the removal rates of total nitrogen.

In order to enrich sufficient amounts of anammox and obtain anammox community with stable nitrogen removal, we adopted a strategy in terms of the gradual increase of NH_4_^+^-N and NO_2_^−^-N levels (Figure 5A) to acclimate the anammox population. The results demonstrated that the NH_4_^+^-N, NO_2_^−^-N, and total nitrogen removal achieved up to 70, 100 and 81%, respectively, within the first 29 days (Figure 5B), when the NH_4_^+^-N and NO_2_^−^-N levels were both controlled at ∼100 mg L^−1^. At the initial stage, the exogenous oxygen in the reactor resulted in the high residual of NO_2_^−^-N and NO_3_^−^-N, suggesting the existence of nitrification and inhibition of anammox. As the remaining oxygen was exhausted, the anammox process turned out to be the major reaction in the reactor. It is worth noting that the nitrogen removal decreased sharply at Day 9, yet it gradually recovered to normal status, and the nitrogen was stably removed subsequently. It was due to the casual change in the influent, whereas the following anammox process was not affected. The simultaneous removal of NH_4_^+^-N and NO_2_^−^-N (ratio of ∼1:1.3) at Day 29 was considered as a mark of anammox performance, and thus the anammox reactor was launched successfully.

Then, the NH_4_^+^-N and NO_2_^−^-N levels were both elevated to ∼200 mg L^−1^ to further acclimate the anammox performance at Day 31 (Figure 5A) in the anammox reactor. After subsequent 18 days of acclimation, the NH_4_^+^-N, NO_2_^−^-N, and total nitrogen removal rates reached up to 87, 100, and 86%, respectively (Figure 5B), and the nitrogen removal ability was equal to that at the previous stage. At Day 51, the NH_4_^+^-N and NO_2_^−^-N levels were further increased to ∼300 mg L^−1^ (Figure 5A). It was found that the NH_4_^+^-N removal had unexpectedly declined, resulting in a sharp decrease in total nitrogen removal (Figure 5B), although the effluent concentrations of NO_2_^−^-N and NO_3_^−^-N were almost unaffected. Afterward, the NH_4_^+^-N removal gradually increased and ranged between 68 and 76% at Day 59–67 (Figure 5B). During this period, the total nitrogen removal rate was maintained between 77 and 83%, and the NO_2_^−^-N removal was nearly close to 100% (Figure 5B). The duration time to achieve total nitrogen removal to more than 80% turned out to be 29, 18, and 8 days, respectively, when the ammonium and nitrite levels both ranged from 100 to 200, and 300 mg L^−1^. The gradually shortened time period also indicated that the nitrogen removal performance of anammox was enhanced.

In the previous study (Manser et al., 2016), fresh anammox granules from cold storage (4°C) were pre-acclimated to 25°C *via* a water bath for about 2 h, which was then shifted into a PSBR containing mixed algal–nitrifying bacterial consortium. Differently, the inoculated anammox in this study was domesticated indoor and preserved long in the lab under optimal conditions. Moreover, the enriched anammox bacteria were also acclimated through stepwise increase of ammonium and nitrite levels to further reinforce their performance before inoculation into PN reactors.

### Startup of Algammox Biofilm System

**FIGURE 6:**
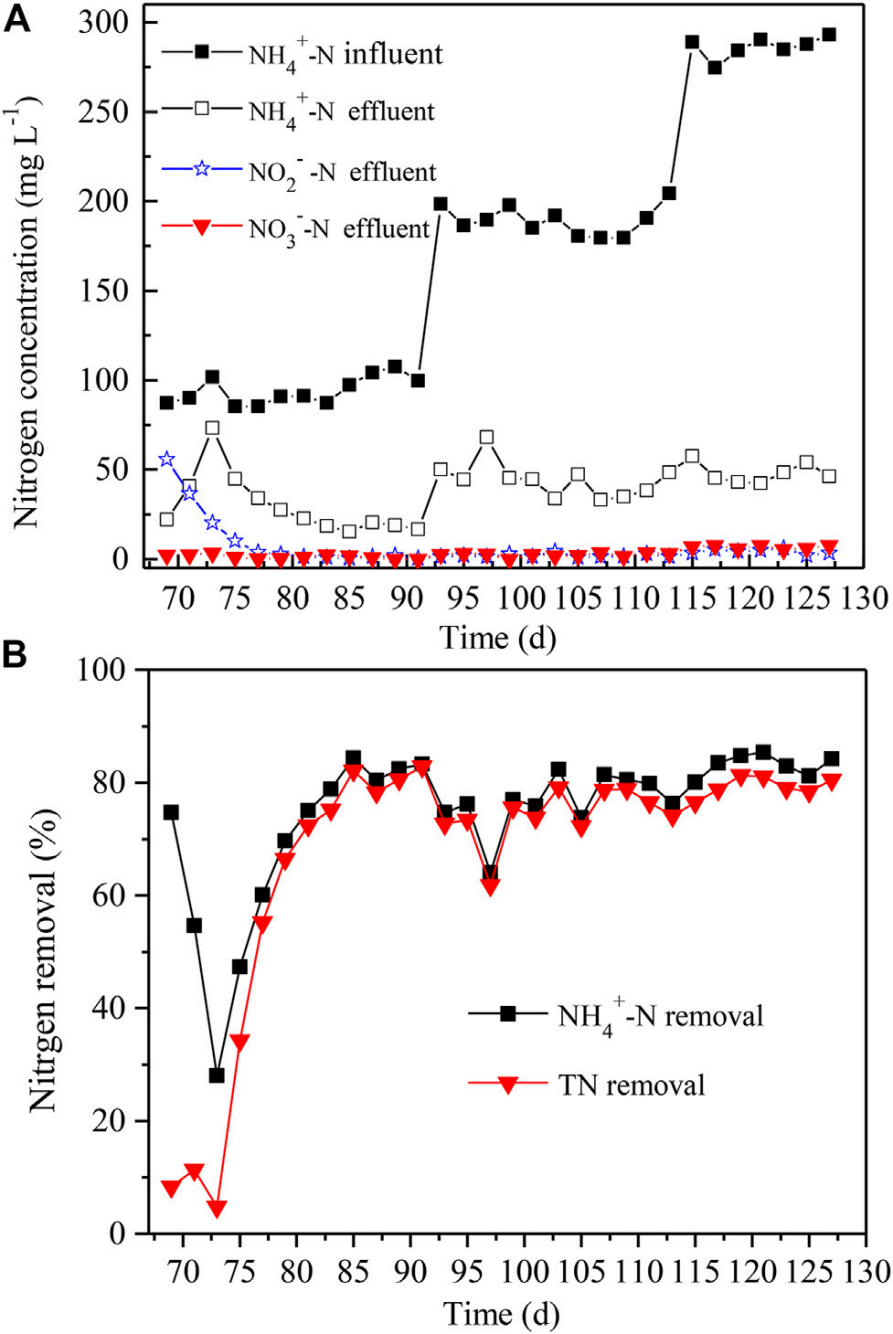
The effluent **(A)** and nitrogen removal **(B)** in microalgae-intensified PN/A reactor with distinct NH_4_^+^-N and NO_2_^−^-N concentrations of influent. The NH_4_^+^-N and NO_2_^−^-N concentrations varied from 100, to 200, to 300 mg L^−1^. TN removal denotes the removal rates of total nitrogen.

The enriched and acclimated anammox were inoculated into the algae-assisted PN reactor without change of DO levels and other parameters. Only NH_4_^+^-N was added as the nitrogen in the simulated sewage, and the initial concentration was set as 100 mg L^−1^. At day 69–73, the NH_4_^+^-N level increased 2.3-fold, and the NO_2_^−^-N level was decreased by 63% in the effluent (Figure 6A). In addition, the NO_3_^−^-N level slightly varied at low levels, i.e., 2–3 mg L^−1^. Afterward, both the NH_4_^+^-N and NO_2_^−^-N levels gradually declined, while the NO_3_^−^-N level was kept stable, all below 2 mg L^−1^. Since Day 81, the NH_4_^+^-N and NO_2_^−^-N levels also began to be maintained at <25 and 2 mg L^−1^, respectively. Until Day 91, the total nitrogen removal and NH_4_^+^-N removal both reached up to 83% (Figure 6A).

After the algammox biofilm system achieved a balance in nitrogen removal, the NH_4_^+^-N levels were further elevated to 200 and 300 mg L^−1^ for the next 18 and 14 days, respectively (Figure 6A). It is exciting that the total nitrogen removal and NH_4_^+^-N removal reached up to 81 and 84% at Day 127, respectively (Figure 6B). During this period (Day 93–127), the NO_2_^−^-N and NO_3_^−^-N levels remained below 6 and 8 mg L^−1^, respectively (Figure 6A). Thus, the microalgae, AOB, and anammox in the reactor all grew and propagated at a steady status, holding a high nitrogen removal. It was potently revealed that the algammox biofilm system could efficiently remove high levels of nitrogen without exogenous supply of oxygen, which was more cost-effective.

**FIGURE 7:**
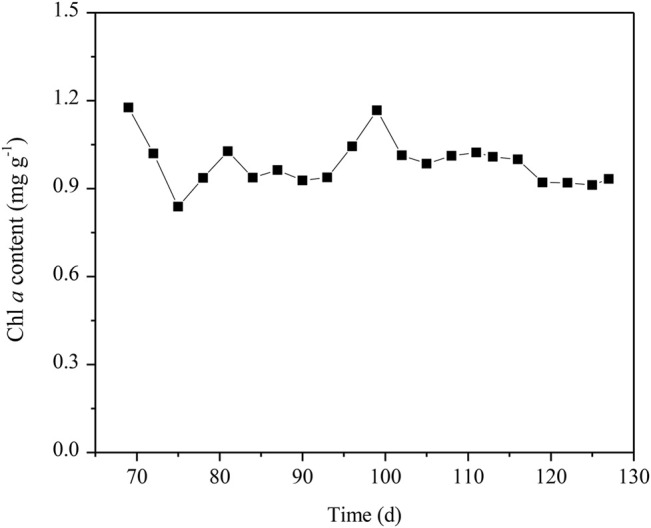
Chl *a* content in the algae-intensified PN and algammox reactors.

After the anammox was inoculated into the algae-assisted PN reactor, the Chl *a* content exhibited an unstable fluctuation between 0.9–1.3 mg g^−1^ at Day 69–127 (Figure 7), suggesting that the algal growth was affected to a certain extent. At Day 127, the DO concentration in the reactor declined to 0.45 mg L^−1^, possibly due to the respiration of inoculated anammox, and the reduced DO levels also inhibited algal growth. Although the algal biomass in the algammox biofilm system was lower than that in the algae-intensified PN reactor, the biological nitrogen removal capacity was not compromised.

**FIGURE 8:**
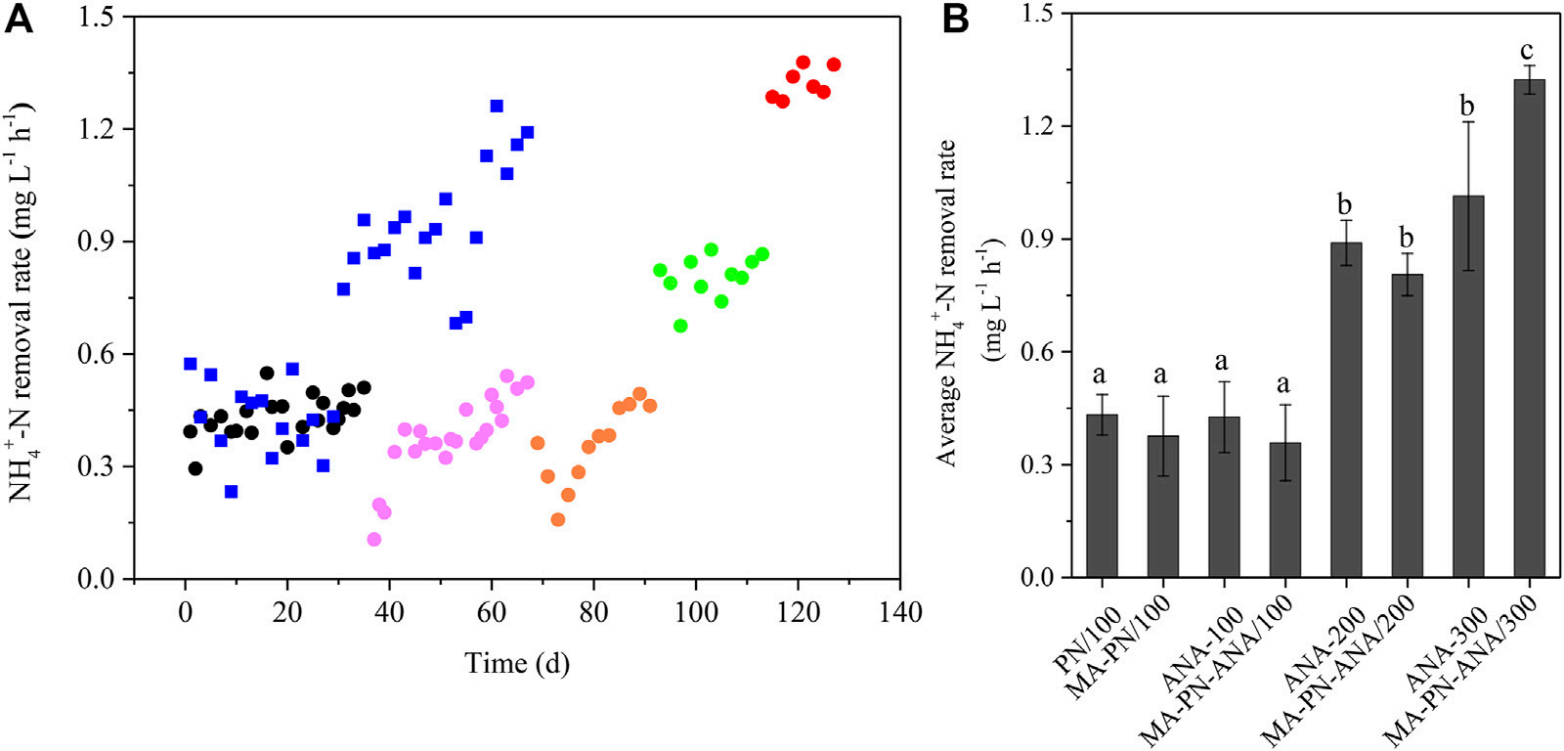
NH_4_^+^-N removal rates at distant stages. **(A)** Black circles denote PN stage at 100 mg L^−1^ of NH_4_^+^-N; pink circles denote algae-intensified PN stage at 100 mg L^−1^ of NH_4_^+^-N; orange circles denote algammox stage at 200 mg L^−1^ of NH_4_^+^-N; green circles denote algammox stage at 300 mg L^−1^ of NH_4_^+^-N; and blue squares denote anammox stage during which NH_4_^+^-N levels increased from 100 (Day 1–29), to 200 (Day 31–49), to 300 (Day 51–67) mg L^−1^. **(B)** PN/100 and MA-PN/100 indicate PN stage and algae-intensified PN stage at 100 mg L^−1^ of NH_4_^+^-N. MA-PN-ANA/100, MA-PN-ANA/200, and MA-PN-ANA/300 indicate algammox stage at 100, 200, and 300 mg L^−1^ of NH_4_^+^-N, respectively. ANA/100, ANA/200, and ANA/300 indicate anammox stage at 100, 200, and 300 mg L^−1^ of NH_4_^+^-N, respectively. The average NH_4_^+^-N removal rates for PN/100 (*n* = 23), MA-PN/100 (*n* = 22), MA-PN-ANA/100 (*n* = 12), MA-PN-ANA/200 (*n* = 11), MA-PN-ANA/300 (*n* = 7), ANA/100 (*n* = 15), ANA/200 (*n* = 10), and ANA/300 (*n* = 9) are expressed as mean ± SD. The distinct letters labeled for the average NH_4_^+^-N removal rates indicate the statistically significant difference by Tukey’s HSD test.

The NH_4_^+^-N removal rates in the sequential batch reactors at distinct stages are shown in Figure 8. Both the PN reactor and anammox reactor presented relatively stable NH_4_^+^-N removal rates, i.e., 0.43 ± 0.07 mg L^−1^ h^−1^ (*n* = 38), when the NH_4_^+^-N level was 100 mg L^−1^. A sharp decline and subsequently gradual increase of NH_4_^+^-N removal rates were observed for the algae-intensified PN reactor and algammox reactor, also when the NH_4_^+^-N level was 100 mg L^−1^. However, the end rates for both reactors reached 0.5 mg L^−1^ h^−1^ on Day 67 and 91, close to that of the PN reactor and anammox reactor. In addition, the NH_4_^+^-N removal rates in the anammox and algammox reactors both increased 2-fold, when the NH_4_^+^-N levels increased from 100 to 200 mg L^−1^. As the NH_4_^+^-N level further increased from 200 to 300 mg L^−1^, the anammox and algammox reactors exhibited 14 and 64% increases in NH_4_^+^-N removal rates, respectively. More importantly, the algammox reactor exhibited more superior performance in NH_4_^+^-N removal (1.32 mg L^−1^ h^−1^), a 31% higher rate, compared to the anammox reactor, when the NH_4_^+^-N level increased to 300 mg L^−1^.

**FIGURE 9:**
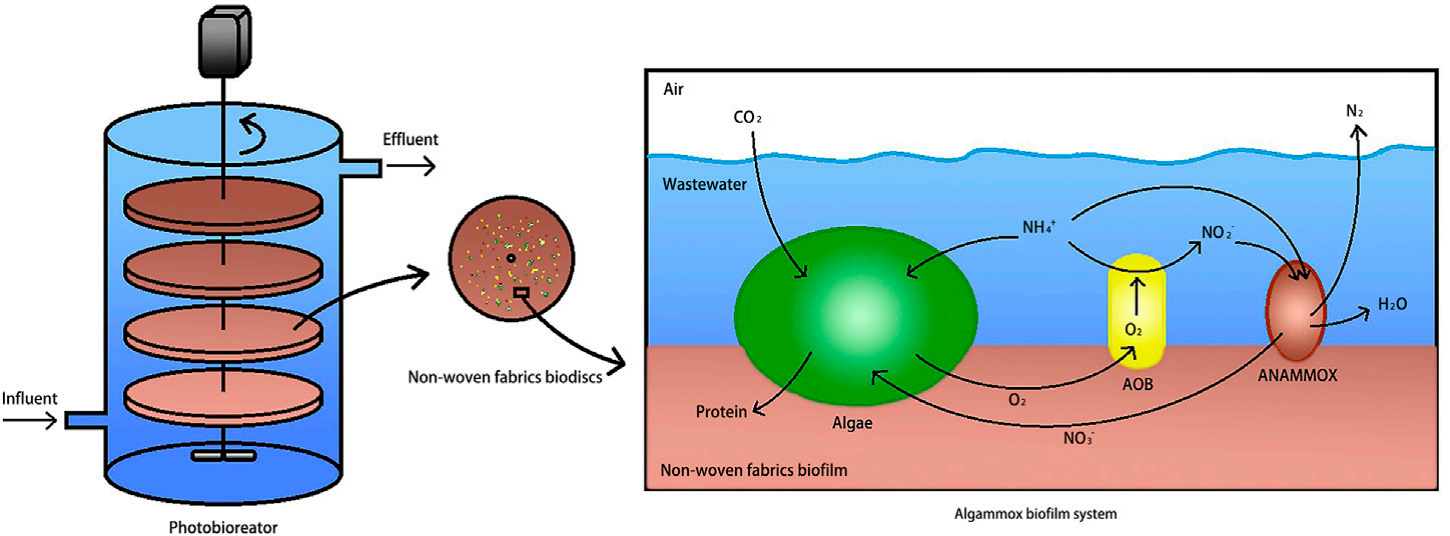
Operation of algammox biofilm system in the photosequencing batch reactor.

In the previous study (Manser et al., 2016), the NH_4_^+^-N removal rate was evaluated to be 5 mg L^−1^ h^−1^ during the illuminated period in a PSBR (0.2 mg L^−1^ of DO and 109 μmol m^−2^ s^−1^ of light intensity). Wang et al. (2015) found that the NH_4_^+^-N removal rates varied between 4.1 and 5.7 mg L^−1^ h^−1^ (0.5 mg L^−1^ of DO and 75 μmol m^−2^ s^−1^ of light intensity) when dealing with high-strength ammonia effluent from an anaerobic digester treating swine manure. In addition, another algal–bacterial consortium achieved an NH_4_^+^-N removal rate as high as 7.7 mg L^−1^ h^−1^ at continuous illumination and much higher DO level of 12 mg L^−1^ (Karya et al., 2013). On the one hand, these distinctions appeared to result from differences in the actual illumination as well as the concerned key factor of DO. The parameter HRT was also considered as another crucial factor. In this study, the HRT was set as 24 h, much shorter than the 4 days in the previous work of Manser et al. (2016). On the other hand, the lower NH_4_^+^-N removal rate in this study was more likely attributed to the relatively less biomass of the functional microalgal–bacterial consortia in the reactors. Even so, the NH_4_^+^-N removal performance indeed got enhanced drastically when the NH_4_^+^-N level gradually increased, which at least demonstrated the feasibility and plasticity of the algammox biofilm system (Figure 9).

### Microbial Community Structure

**FIGURE 10:**
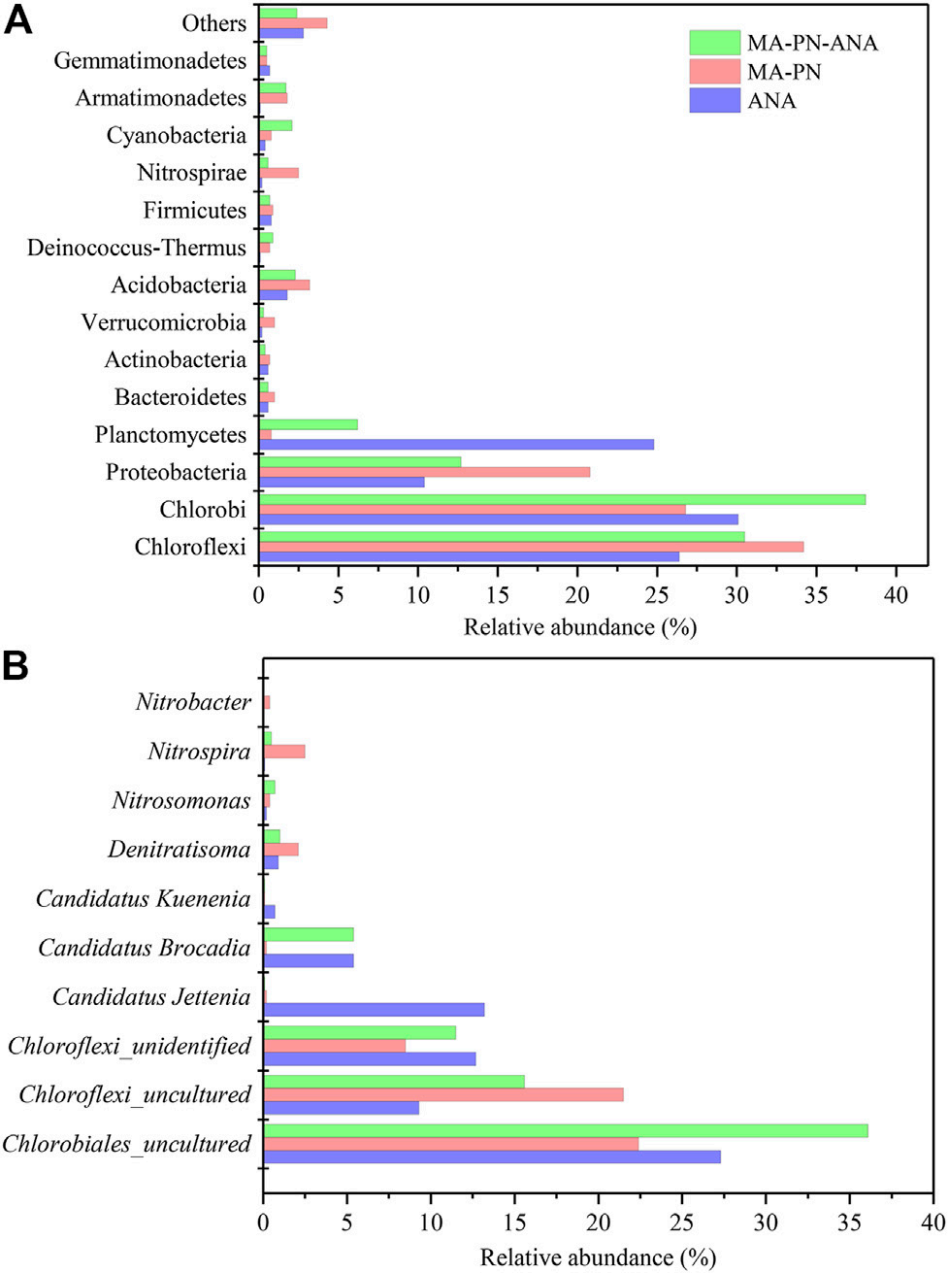
The relative abundance of dominant functional bacteria at phylum **(A)** and genus **(B)** levels in three reactors. ANA, MA-PN, and MA-PN-ANA denote anammox reactor, microalgae-intensified partial nitrification reactor, and algammox reactor, respectively.

A total of 21 phyla were detected from the three samples, i.e., ANA, MA–PN, and MA-PN-ALA. Among those, the phyla with the highest relative abundances included Chloroflexi, Chlorobi, Proteobacteria, and Planctomycetes (Figure 10). Commonly, the phyla Chloroflexi, Chlorobi, Proteobacteria, Bacteroidetes, and/or Candidate divisions (the uncultured heterotrophic bacteria) could coexist with anammox bacteria (Cho et al., 2010; Kindaichi et al., 2012). In this study, both the relative abundances of Chloroflexi and Chlorobi achieved up to more than 25% in the aforementioned three reactors, hinting at the potentially vital roles of these bacteria in nitrogen removal. The coexisting Chloroflexi were revealed to be capable of scavenging organic matters originated from anammox bacterial cells in anammox reactors (Kindaichi et al., 2012). The heterotrophic Chlorobi were also found to be dominant in the microbial community of PN and complete nitrification processes (Zhao et al., 2018). Bacteria belonging to Chlorobi were shown to be highly vibrant protein degraders and could decompose extracellular peptides of anammox granules in a laboratory-scale bioreactor (Lawson et al., 2017). Beyond this, the functional bacteria taking part in nitrogen removal included Proteobacteria, Planctomycetes, and Nitrospirae (Mai et al., 2021). The anammox belonged to Planctomycetes, the AOB and certain NOB belonged to Proteobacteria, and certain NOB, e.g., *Nitrospira*, belonged to Nitrospirae.

The relative abundance of Proteobacteria was the highest in the MA-PN reactor (22%), followed by the MA–PN–ANA (14%) and ANA (11%) reactors, respectively. In addition, the relative abundance of Planctomycetes turned out to be the highest in the ANA reactor, i.e., 24%, while the MA–PN–ANA and MA–PN reactors contained 6% and below 1% of this phylum, respectively. For Nitrospirae, its propagation was inhibited, to a large extent, and its relative amounts in the three reactors were all below 2.5%. In view of bacterial profiles, the functional bacteria for nitrogen removal in the ANA reactor were mainly Planctomycetes, performing anammox reaction, while in the MA–PN reactor, these were mainly Proteobacteria, typically dominated by AOB, and in the MA–PN–ANA reactor, these included mainly both Planctomycetes and Proteobacteria, in which the biological nitrogen removal was successful .

The typical bacteria taking part in ammoxidation mainly include *Nitrosomonas* (belonging to Proteobacteria) and that taking part in nitrification mainly include *Nitrospira* (belonging to Nitrospirae) and *Nitrobacter* (belonging to Proteobacteria). *Denitratisoma* was the bacteria conducting denitrification, and the dominant genera carrying out anammox reaction consist of *Candidatus Kuenenia*, *Candidatus Jettenia*, and *Candidatus_Brocadia* (Wang et al., 2019). Figure 10B shows the relative abundances of these functional bacteria for nitrogen removal. The dominant bacteria in these three reactors belonged to phyla Chlorobi and Chloroflexi, either uncultured or unidentified species, and their total abundance accounted for more than 50%, suggesting that they might be the potential functional bacteria closely correlated to nitrogen removal (Cho et al., 2010; Kindaichi et al., 2012; Lawson et al., 2017; Zhao et al., 2018). *Nitrosomonas*, *Nitrospira*, *Nitrobacter*, and *Denitratisoma* all occurred in the MA-PN reactor, and their relative amounts were determined to be 0.4, 2.5, 0.4, and 2.1%, respectively. It is surprising that the amounts of nitrifier and denitrifier were much higher than that of AOB. It was presumed that the introduction of microalgal cells increased the amounts of organic matters, causing the occurrence of denitrifiers. However, the denitrifiers were postulated to conduct partial denitrification and convert NO_3_^−^-N to NO_2_^−^-N (You et al., 2020), which made NAR reach up to more than 90% when AOB was not so abundant.

The dominant bacteria in the anammox reactor were detected to be *Candidatus Jettenia*, *Candidatus Brocadia*, and *Candidatus Kuenenia*, and their abundance achieved up to 13.2, 5.4, and 0.7%, respectively. They co-functioned to maintain effective ammonia removal and nitrite accumulation. The MA–PN–ANA reactor was operated with the aid of the key roles of *Candidatus Brocadia* (5.4%), *Denitratisoma* (1.0%), *Nitrosomonas* (0.7%), and *Nitrospira* (0.5%). In addition, the NOB (*Nitrospira* and *Nitrobacter*) and denitrifier (*Denitratisoma*) levels greatly declined by 83 and 52%, respectively, while the AOB (*Nitrosomonas*) level increased by 75%, when anammox were inoculated into the MA–PN reactor. Moreover, the dominant anammox species varied from *Candidatus Jettenia* in the anammox reactor to *Candidatus Brocadia* in the MA–PN–ANA reactor. Thus, the inoculated anammox seemed to interact with the original functional bacteria in the MA-PN reactor responsible for nitrogen removal, accelerating the formation of optimized microbial structures. In this case, the ammoxidation and anammox reaction were greatly reinforced, and nitrification together with denitrification was largely inhibited, accompanied by a shift of anammox species. Besides, the dominance of *Candidatus Brocadia* also occurred in single-stage partial-denitrification and anammox systems, emphasizing its highly competitive trait in relation to other anammox genera (Du et al., 2020; Wang et al., 2020; Xu et al., 2020). It was demonstrated that the gradually stable running of PN and anammox reactions depended on the cooperation of the algal cells and potential functional bacteria (Supplementary Figure S4), assuring the effective biological removal of nitrogen in the MA-PN-ANA reactor as illustrated in Figure 9.

## Conclusion

Partial nitrification and anammox system based on an algal–bacterial consortium, i.e., algammox biofilm system, was successfully launched in a PSBR within 127 days. The key DO parameter was strictly controlled at <0.6 mg L^−1^ dependent on the available light cycles, i.e., 8 h light:16 h dark. The ammonia removal rates were notably elevated, attributing to high-performance reinforcement *via* the stepwise increase of ammonia levels as well as the co-function of microalgae, AOB, and anammox. The successful startup of the algammox biofilm system was ascribed to the enhanced roles of AOB (*Nitrosomonas*) and anammox (*Candidatus Brocadia*), while the NOB (*Nitrospira* and *Nitrobacter*) and denitrifier (*Denitratisoma*) were found to be inhibited greatly. Collectively, the algammox biofilm system could run for a long time to treat high-ammonia wastewater under controllable DO levels, regulated by photosynthetic algal cells, without the addition of either energy intensive aeration or external organic carbon.

## Data Availability

The original contributions presented in the study are included in the article/**Supplementary Material,** further inquiries can be directed to the corresponding author.
