# Supplementary material for: Achieving Partial Nitrification-Anammox Process Dependent on Microalgal-Bacterial Consortia in a Photosequencing Batch Reactor
Source: Front Bioeng Biotechnol. 2022 Mar 18;10:851800. doi: 10.3389/fbioe.2022.851800 (PMC8971602; doi:10.3389/fbioe.2022.851800)
Supplement: Supplementary file 1 [file DataSheet1.pdf]

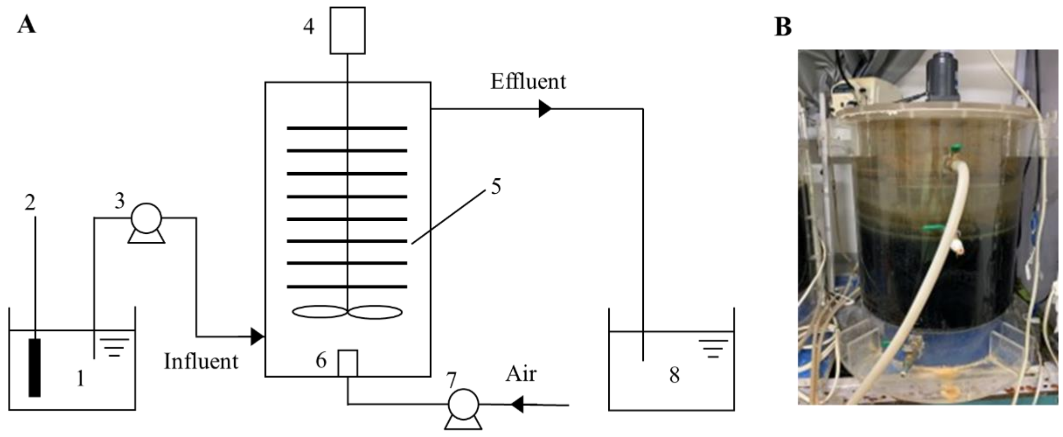

Fig. S1. Schematic diagram (A) and photo (B) of PN reactor. 1-influent tank; 2-heater; 3-peristaltic pump; 4-mixer; 5-non-woven fabrics discs; 6-aerator; 7-air pump; 8-effluent tank.

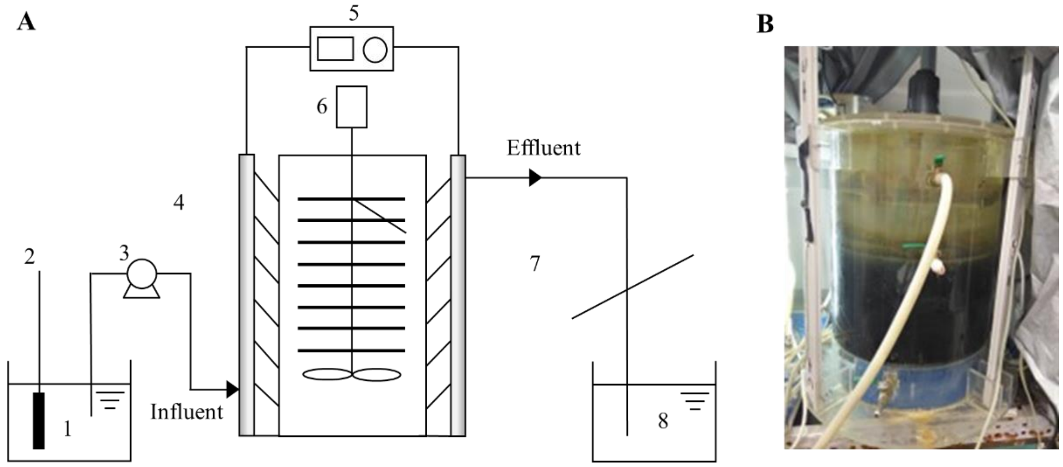

Fig. S2. Schematic diagram (A) and photo (B) of microalgae-partial nitrification reactor. 1-influent tank; 2-heater; 3-peristaltic pump; 4-light tubes; 5-time switch; 6- mixer; 7- non-woven fabrics discs; 8-effluent tank.

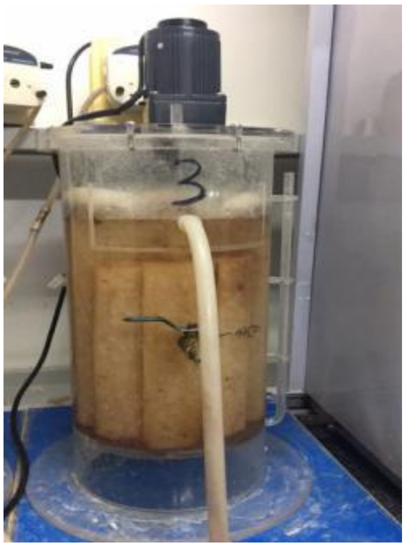

Fig. S3. Anammox reactor with non-woven fabrics materials.

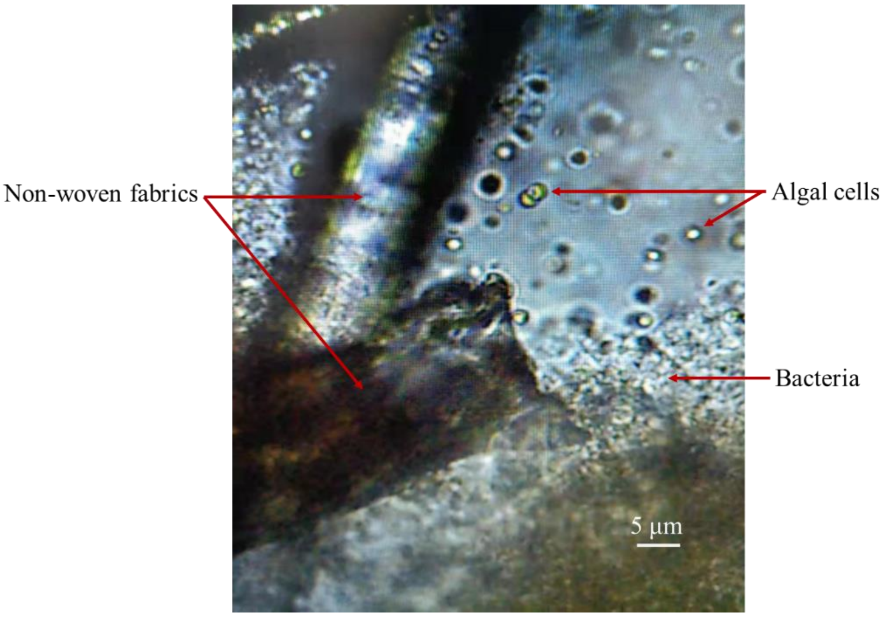

Fig. S4 Algal cells and the potential functional bacteria on non-woven fabrics in the algammox system.
